# Regulation of melanoma initiating cells by Hedgehog signaling and SOX2

**DOI:** 10.1186/1479-5876-12-S1-O4

**Published:** 2014-05-06

**Authors:** Roberta Santini, Silvia Pandolfi, Valentina Montagnani, Silvia Pietrobono, Nicola Pimpinelli, Lorenzo Borgognoni, Barbara Stecca

**Affiliations:** 1 Laboratory of Tumor Cell Biology, Core Research Laboratory - Istituto Toscano Tumori, Florence, Italy; 2 Dept. of Dermatology, University of Florence, Florence, Italy; 3 Plastic Surgery Unit, S.M. Annunziata Hospital - Regional Melanoma Referral Center, Istituto Toscano Tumori, Florence, Italy

## Background

Recent reports suggest that within the heterogeneous population that constitutes a melanoma, certain cell types exhibit molecular and functional features similar to stem cells. These melanoma-initiating cells (MICs) have the ability of unlimited self-renewal, multilineage differentiation and the potential to initiate and maintain tumor growth [1]. Furthermore, MICs are believed to confer chemoresistance to conventional chemotherapeutic agents and newly developed molecularly-targeted drugs [2,3]. Therefore, defining the molecular and biochemical pathways that support MICs is of critical importance for the development of more efficient targeted therapies. We have previously shown that the HEDGEHOG (HH) signaling is required for melanoma growth [4] and for survival and expansion of MICs [5]. Here we investigate the mechanism by which inhibition of the HH signaling leads to a decrease of MIC stemness, addressing the role of the transcription factor SOX2.

## Materials and methods

MICs were enriched by Fluorescence Activated Cell Sorting using the metabolic marker Aldehyde Dehydrogenase (ALDH) and by establishing melanoma spheres in non-adherent culture conditions from 20 human melanomas. Expression of SOX2 and of HH pathway components was determined by real time PCR and Western blot analysis. Modulation of the HH signaling was performed by stable expression of lentiviral vectors encoding short-interference RNAs specific for SMO or GLI1 (to inhibit HH), and PTCH1 (to activate HH). Functional analysis of SOX2 was performed by stable overexpression and silencing using lentiviral vectors.

## Results

We find that the HH signaling regulates the expression of SOX2 and the downstream effectors of the HH signaling, the transcription factors GLI1 and GLI2, bind to *SOX2* promoter in melanoma cells. Functionally, we show that SOX2 is required for HH-induced melanoma cell growth and MIC self-renewal. We present evidence that SOX2 is highly expressed in a population enriched for cancer stem cells in patient-derived melanomas. Knock-down of SOX2 sharply decreases self-renewal of melanoma spheres and of ALDH^high^ melanoma stem cells. Consistently, ectopic expression of SOX2 in melanoma cells is sufficient to enhance stemness *in vitro.* SOX2 silencing also inhibits cell growth and induces apoptosis in melanoma cells. Most importantly, depletion of SOX2 drastically impairs tumorigenicity of ALDH^high^ MICs in orthotopic xenografts.

## Conclusion

Our data identify SOX2 as a novel mediator of the HH signaling in melanoma cells and indicate that SOX2 is a critical factor for the survival of MICs. These findings could provide the basis for novel therapeutic strategies based on the inhibition of SOX2 for the treatment of human melanomas.
